# Improving transcatheter aortic valve interventional predictability via fluid–structure interaction modelling using patient-specific anatomy

**DOI:** 10.1098/rsos.211694

**Published:** 2022-02-09

**Authors:** Vijay Govindarajan, Arun Kolanjiyil, Nils P. Johnson, Hyunggun Kim, Krishnan B. Chandran, David D. McPherson

**Affiliations:** ^1^ Division of Cardiology, Department of Internal Medicine, McGovern Medical School, The University of Texas Health Science at Houston, 1881 East Road, Houston, TX 77054, USA; ^2^ Department of Mechanical and Nuclear Engineering, Virginia Commonwealth University, Richmond, Virginia, USA; ^3^ Department of Bio-Mechatronic Engineering, Sungkyunkwan University, Suwon, Gyeonggi, Korea; ^4^ Roy J. Carver Department of Biomedical Engineering, The University of Iowa, Iowa City, IA, USA

**Keywords:** aortic valve intervention, fluid–structure interaction, patient-specific modelling, interventional predictability

## Abstract

Transcatheter aortic valve replacement (TAVR) is now a standard treatment for high-surgical-risk patients with severe aortic valve stenosis. TAVR is being explored for broader indications including degenerated bioprosthetic valves, bicuspid valves and for aortic valve (AV) insufficiency. It is, however, challenging to predict whether the chosen valve size, design or its orientation would produce the most-optimal haemodynamics in the patient. Here, we present a novel patient-specific evaluation framework to realistically predict the patient's AV performance with a high-fidelity fluid–structure interaction analysis that included the patient's left ventricle and ascending aorta (AAo). We retrospectively evaluated the pre- and post-TAVR dynamics of a patient who underwent a 23 mm TAVR and evaluated against the patient's virtually de-calcified AV serving as a hypothetical benchmark. Our model predictions were consistent with clinical data. Stenosed AV produced a turbulent flow during peak-systole, while aortic flow with TAVR and de-calcified AV were both in the laminar-to-turbulent transitional regime with an estimated fivefold reduction in viscous dissipation. For TAVR, dissipation was highest during early systole when valve deformation was the greatest, suggesting that an efficient valve opening may reduce energy loss. Our study demonstrates that such patient-specific modelling frameworks can be used to improve predictability and in the planning of AV interventions.

## 1. Introduction

Transcatheter aortic valve replacement (TAVR) is a minimally invasive aortic valve (AV) replacement technique by which a new valve is inserted inside the stenosed/diseased valve by means of a catheter. Once in position and expanded from its initial collapsible state, TAVR pushes the native AV and takes over the function of ensuring a unidirectional flow between left ventricle (LV) and aorta. TAVR is now a widely accepted intervention for high-risk patients with severe AV stenosis [1]. TAVR is expanding to treat a wider range of pathology such as degenerated bioprosthetic valves (valve-in-valve), bicuspid valves and for aortic insufficiency [2,3]. This is in part due to encouraging results from evidence-based studies suggesting that the performance of TAVR is comparable to surgical AV replacements in high-risk, but operable patients in terms of mortality, re-hospitalization and quality of life improvement post-intervention [4,5]. In fact, TAVR is being increasingly explored in intermediate- to low-risk patients [6] with studies demonstrating normal valvular function 5 years post-TAVR [7,8]. These broader indications necessitate further study into the long-term haemodynamic performance and durability of TAVR [1].

Several factors play a key role in the immediate success of TAVR and have been quantitatively studied. These include device deployment to prosthesis positioning and their interaction with aortic root which ultimately impact leaflet coaptation [9–13]. While these factors play a structural role in impacting short-to-intermediate outcome of TAVR, haemodynamics plays a significant role in determining both immediate and long-term outcomes of TAVR (and surgical replacements). Long-term performance of AV prosthesis is evaluated by quantitative parameters such as peak velocity, AV mean pressure gradient, valve area and Doppler velocity index [14]. Overall, cardiac function is typically assessed using stroke volume, ejection fraction and cardiac output. These parameters heavily depend on the local fluid dynamics that in turn depend on the valvular dynamics including leaflet deformation, valve opening and closing times and the effects of the native (and calcified) aortic leaflets on post-implantation TAVR orientation.

Previous studies suggest that substantial improvement can be achieved in terms of transvalvular systolic gradient and flow, AV area and cardiac output post-TAVR [15,16]. However, it is difficult to quantify how these post-TAVR parameters and flow dynamics compare with a structurally normal native AV as these data are not available. Such a comparison would help predict TAVR's ability toward restoring normal LV and aortic flow. It is also challenging to predict the valve size, design type or its orientation that would produce the most-optimal flow profile for each patient. The ability to quantitatively compare these parameters against normal AVs serving as a patient-specific benchmark can help in better planning of TAVR to potentially improve long-term success for the patient.

Fluid–structure interaction (FSI) modelling offers the capability to simultaneously predict both post-op AV dynamics and its associated blood flow. The current state-of-the-art in FSI-based AV studies use a conduit-type inlet and outlet to represent ventricular inlet and aortic outlet, respectively [17–22]. Although these studies have provided insights into the AV dynamics and associated blood flow in the immediate vicinity of the valve leaflets [17–24], application of a conduit-type inlet to represent LV wall motion-induced change in LV pressure may affect the overall accuracy of the computation. Additionally, it may be a challenge to impose patient-specific boundary conditions using models with simplified conduit-type ventricular inlets and, hence, may not be most suitable for patient-specific planning and evaluation of AV interventions.

Peak systolic flow in the aorta is known to be in the laminar-to-turbulent transitional flow regime and can go up to a turbulent Reynolds number (*Re*) of 10 000 under disease conditions [25]. Under such high *Re*, flow patterns could involve high magnitudes of jet flow, strong boundary layer separation, vortex formation and shedding [22,26]. A high-density mesh is required to accurately resolve such complex flow dynamics. Combined with a highly nonlinear and instantaneous valve deformation, simulation time for an FSI model at physiological *Re* could be prohibitively long for pre-operative planning of AV interventions. Such challenges can either force us to use a coarse mesh [21] or use other artificial methods such as higher-than-physiological viscosity [22], truncate the computational domain into conduit-type models [17–20,22] or restrict the analysis to two-dimension [23,24], hence potentially compromising physiological accuracy. However, the implications of the AV function extend far beyond the vicinity of the leaflets [27]. Including the LV and its systolic contraction and aortic geometry in the analysis allow comprehensive and realistic evaluation of systolic AV function.

**Figure 1.**
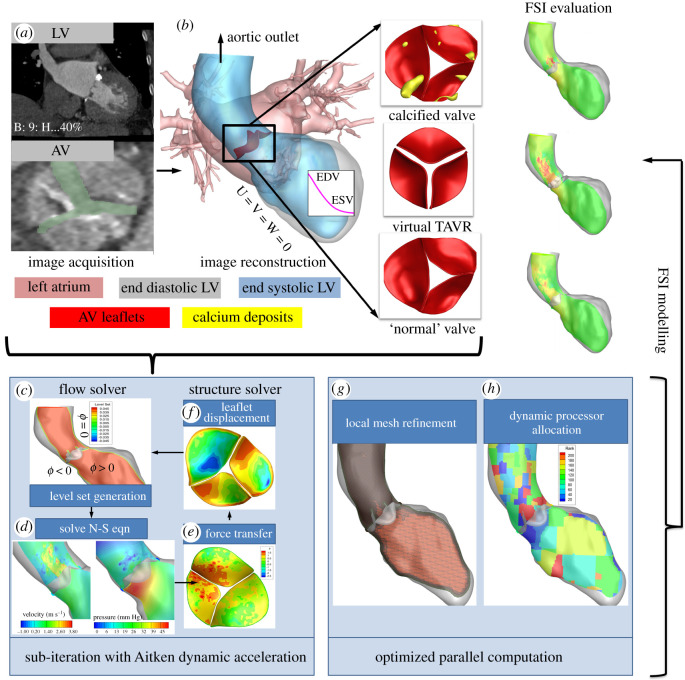
Patient-specific workflow for predictive evaluation of TAVR. (*a*) Three-dimensional patient-specific models are constructed from CT images acquired during the standard clinical protocol for TAVR. (*b*) FSI-based evaluation protocol for assessing performance of TAVR against SAV and de-calcified normal AV. Also shown are the boundary conditions for flow: no-slip and no-penetration conditions are imposed at the wall. Fluid volume displaced by the moving interfaces (LV contraction and AV leaflet motion) are computed and applied as outflow at the aortic outlet [33]. Please note that aortic outlets were extended to avoid numerical instabilities (not shown). (*c*–*h*) Workflow for image-based fluid–structure interaction solver equipped with local adaptive mesh refinement and dynamic partition strategy.

In this study, we extended our previously validated FSI algorithm combined with patient-specific imaging towards improving the current state-of-the-art in predictive evaluation of AV interventions. To that end, we included (i) systolic contraction of LV walls based on the patient's heart rate; (ii) high-fidelity flow computation with adaptive meshing (approx. 15–20 million) with flow-based local mesh refinement (LMR) for accurate resolution of high *Re* flows; (iii) dynamic partitioning strategy for efficient computation; (iv) implementing enhanced assumed solid-shell (EAS) element routine for discretizing leaflet geometries (approx. 8000 eight-node hexahedral elements/leaflet) that are known to have superior accuracy [28,29], and (v) Fung-type hyperelastic material model to accurately capture AV dynamics during FSI computation [30–32]. Our patient-specific AV evaluation workflow and algorithmic/modelling framework are pictorially illustrated in figure 1 and its details explained in §2. Using our modelling framework, we retrospectively predicted pre- and post-TAVR LV and AV dynamics for a patient suffering from a severely stenotic AV who received a 23 mm TAVR. The main focus of our evaluation was to quantitatively compare post-TAVR dynamics with that of diseased AV dynamics to predict the degree of improvement achieved post-intervention. Uniquely, we virtually removed the calcium deposits from the AV leaflet surfaces identified via computed tomography (CT) images to predict the patient's hypothesized normal AV dynamics that served as a patient-specific benchmark during our evaluation process. To the best of our knowledge, such a patient-specific modelling framework or an evaluation workflow has not been realized previously. Model-predicted peak systolic velocity, pressure gradients, valve opening dynamics, LV pressure distribution, wall shear stress (WSS) on LV and ascending aorta (AAo), vorticity and viscous dissipation (energy loss) were quantitatively evaluated to comprehensively assess valvular performance.

## 2. Methods

This retrospective study of anonymous haemodynamic tracings and CT images acquired as part of routine clinical care was approved by the institutional review board without the need for additional informed consent.

The patient-specific workflow for evaluating AV described in this study was extended from our previously validated FSI algorithm tailored for mitral valve simulations [34]. Figure 1 illustrates our new AV evaluation workflow with its methodology described in the following sections. Briefly, three-dimensional models of AV, LV (end diastole) and the aortic root were segmented and reconstructed from de-identified CT data acquired as part of routine clinical care for TAVR from a 70-year-old woman with severe symptomatic AS who underwent implantation of a 23 mm Edwards Sapien device. To simulate systolic LV function, the LV walls were prescribed with a contraction rate based on the patient's heart rate of 70 bpm, assuming a systolic duration of 1/3 of the cardiac cycle. The resulting increase in LV pressure created a gradient across the AV to which the leaflets deformed. The mitral valve was assumed to be fully closed during systolic ejection and hence not included in the computation. Our comparative evaluation included the patient's pre-op stenosed AV (SAV), the patient's virtually decalcified ‘normalized’ AV (NAV) that served as a patient-specific benchmark and finally, a virtually implanted 23 mm TAVR (figure 1).

### 2.1. Image acquisition and three-dimensional model reconstruction of patient LV, AV, TAVR and Ao

The AV, LV and aortic root were reconstructed from de-identified patient-specific CT data (figure 1*a,b*). The patient's scan consisted of images taken at 10 distinct phases of the cardiac cycle. For extracting the geometries, 3D slicer, an open-source tool for digital imaging and communications in medicine file, was used as the segmentation tool [35]. First, the images of peak diastole were identified and imported into 3D slicer. The 3D slicer segmentation module has a basic set of algorithms to segment images of different modalities. A semi-automated segmentation algorithm based on thresholding and region growing was used to segment the AV, LV and aortic root [36]. The segmentation algorithm transformed the voxels/pixels into objects, and the delineated voxels were used to create a three-dimensional rendering of each object. With the same threshold parameters, rough segmentation using segment editor, paint and smoothing functions was repeated to modify the objects until sufficiently smooth meshes were obtained. The surface rendering for each object was exported from 3D slicer in the stereolithography format and refined using a computer-aided design (CAD) software package. At this stage, the three-dimensional model for each object was further refined and smoothened to remove noise artefacts from the segmentation thresholding method [37].

The three-dimensional model of 23 mm TAVR leaflets was constructed using the commercial CAD software package GAMBIT (ANSYS, Canonsburg, PA). The TAVR was virtually inserted in the place of native AV. In the current study, presence of the native calcified AV pushed aside by the TAVR was assumed to be a non-moving entity occupying space between the TAVR housing and the aortic walls. Thus, the non-moving native AV acted as walls (no slip and no penetration) and was integrated with the aortic walls.

### 2.2. Simulation of left ventricular wall contraction

In this study, the AV leaflets responded to a rise in LV pressure (consequently pressure drop across AV) due to the contraction of LV. This was achieved by prescribing a LV wall motion tuned to the patient's heart rate, in effect producing a rise in LV pressure as walls contracted inwards. The initial configuration of the LV was the patient's reconstructed model imaged at end-diastole that moved inwards to the end-systole based on the motion specified by our LV contraction model.

In our LV contraction model, spatial changes in the contraction rate or the non-uniformity in LV wall contraction, particularly from base to apex, and that between septal and lateral walls were controlled by means of a Gaussian function of the form in each of the *x*, *y* and *z* coordinates

2.1 $${\left|\;f(x,\;t)=a\;.\;\exp\left(-\frac{{\left(x-b\right)}^{2}}{2c^{2}}\right)\right|}_{x,y,z}.$$

In equation (2.1), *f*(*x*, *t*) represents the spatial contraction rate applied along the LV walls that varies in space and time; *a* represents rate of contraction, which is based on a typical systolic duration (1/3 of cardiac cycle), patient's heart rate and end diastolic to end systolic volume; *b* and *c* are tuned constants that govern the centre and peak of the Gaussian function for each of the *x*, *y*, *z* directions (*b* (*x*, *y*, *z*) = −1.4, −1.6, −2.6 and *c* (*x*, *y*, *z*) = 0.58, 0.5, 0.8) such that septal wall motion is limited to moving inwards in the normal direction, while the lateral walls contract in all three directions, thus making the LV contraction realistic effecting a change in LV systolic flow dynamics.

### 2.3. Patient-specific FSI modelling

Our FSI algorithm couples a computational fluid dynamics (CFD) solver and a finite-element (FE) structural solver to simultaneously solve both blood flow (fluid), LV wall contraction and leaflet dynamics (FE) [34], thus representing a comprehensive and realistic approach to simulate/predict AV function, LV and aortic flow. In this subsection, we provide a brief overview of the algorithmic components that has been previously developed and validated.

Our partitioned coupling involves a validated massively parallel CFD solver using a Eulerian level set-based fixed Cartesian grid based on a hybridized ghost fluid method to solve the incompressible Navier–Stokes equation (equations (2.2) and (2.3)) [34,38–41],

2.2 ∇⋅u=0

and

2.3 $$\frac{\partial u}{\partial t}+u\cdot \nabla u=-\Delta p+\frac{1}{Re}\nabla^{2}u.$$

In equations (2.2) and (2.3), ***u*** denotes fluid velocity vector, *p* represents the fluid pressure and *Re* = *ρVD*/*μ* denotes the Reynolds number, in which *ρ*, *V*, *D* and *μ* refer to fluid density, characteristic flow velocity, length and fluid viscosity, respectively. Details of the hybridized ghost fluid method, spatial and temporal discretization schemes used in our flow solver along with the comprehensive validation studies are described in [39].

The moving interfaces, namely AV leaflets and LV walls, were implicitly represented by level set fields. Level set fields (*ϕ*) are signed normal distance functions from a point where flow is solved in the cells with positive values and the zero-level set (*ϕ* = 0) contour representing the immersed boundary (figure 1*c*) where the boundary conditions are applied/exchanged during computation [34]. Combined with the fixed Cartesian grid, this framework allows an easy representation of complex geometries and their motion within the computational mesh without the need for constant remeshing [34,38,39]. The motion of the level set field is governed by the advection equation (equation (2.4)), where ***V*** is the level set propagation velocity driven by the physics of the problem.

2.4 $$\frac{\partial \phi }{\partial t}+V\cdot \nabla \phi =0.$$

The pressure and the shear force computed on the AV leaflet surfaces (immersed interface) by the CFD solver (figure 1*d*,*e*) are passed to the structural solver as loading conditions. These fluid forces will elicit an instantaneous response on the thin and pliant valve leaflets having high nonlinear material properties [34,38]. To capture the resulting high rates of AV leaflet deformation during LV systolic ejection, we used an EAS element routine with displacement degrees of freedom, superior bending accuracy and free of shear or volumetric locking [28,29] making it ideal for heart valve leaflet modelling. This was achieved in our earlier studies with mitral valve where EAS element accurately predicted the highly nonlinear behaviour of mitral valve under physiological loading conditions [34]. In this study, the AV leaflets were represented by a hyperelastic Fung material model which has been extensively used in the past to model AV dynamics [30,31,42,43]. For the Fung material model, the second Piola–Kirchhoff stress tensor is given by the relationship (equation (2.5)),

2.5 $$S=\frac{\partial W}{\partial E}.$$

In the above equation, *W* is the hyperelastic strain energy and *E* is Green–Lagrangian strain. The strain energy, *W*, is established by an exponential form (equation (2.6)),

2.6 $$W=\frac{c}{2}[e^{Q}-1],$$

where *c* is a material constant, and *Q* is given by (equation (2.7)),

2.7 $$Q=A_{1}E_{11}^{2}+A_{2}E_{22}^{2}+A_{3}E_{11}E_{22}+A_{4}E_{12}^{2}+2A_{5}E_{11}E_{22}+2A_{6}E_{22}E_{12},$$

where *E_ij_* are Green–Lagrangian strain components, and *A*_1−6_ are material constants. The values of *C*, *A*_1−6_ for each of the valve types used in the current study were obtained from previous studies [30–32]. To simulate the effect of increased leaflet stiffness due to severe calcification for SAV, we assumed a 10-fold increase in the value of *C* with a uniform distribution based on previous studies [23,24]. The structural solver solves for displacement (figure 1*f*), and Newmark algorithm was used to compute leaflet velocity and acceleration [34]. This information is passed on to the fluid solver to update the position and velocity of the zero-level set contours (figure 1*c*). The interface velocity is then extended along in the normal direction to define the propagation velocities elsewhere in the fluid computational domain [39].

The coupling of fluid and solid subdomain was enforced strongly at the valve leaflet interface [34] denoted by *Γ_fs_* in equation (2.8) with kinematic (equation (2.8*a*–*c*)) and dynamic (equation (2.8*d*)) matching conditions to ensure continuity in position and traction forces. It should be noted that, for the fluid subdomain, it is the zero-level set contours for each of the leaflets.

2.8*a* $$\phi (x,\;t)=0=x_{s}|\Gamma_{\;fs},$$

2.8*b* $$u_{f}|\Gamma_{\;fs}={\dot{x}}_{s}|\Gamma_{\;fs},$$

2.8*c* $$a_{f}|\Gamma_{\;fs}={\ddot{x}}_{s}|\Gamma_{\;fs}$$

2.8*d* $$\mathrm{and}\;\sigma_{s}|\Gamma_{\;fs}\cdot n=\sigma_{f}|\Gamma_{\;fs}\cdot n.\;$$

In the above equations (equation (2.8*a*–*c*)), ϕ(x, t)=0 denotes the zero-level set, ***x_s_*** denotes the position of the interface, while ***u_f_*** and ***a_f_*** refer to fluid velocity and acceleration, respectively. In equation (2.8*d*), *σ* represents the stress tensor and ***n*** is the unit normal to the FSI interface *Γ_fs_*.

The fluid and the solid subdomains were strongly coupled at the FSI interface by means of sub-iterations until convergence (tolerance = 1.0 × 10^−6^) to counteract the numerical instabilities due to added mass effect [44–46] induced by a solid-to-fluid density ratio approximately 1 that is typical for physiological heart valve FSI simulations [34]. Furthermore, a dynamic Aitken under-relaxation method [44,45] which was previously implemented in our sub-iteration scheme [34] was used in this study to accelerate the FSI convergence. Our FSI algorithm was previously validated [34] against standard ‘heart valve inspired’ benchmark studies involving strong added mass effect [47–49].

### 2.4. Local mesh refinement and adaptive repartitioning strategy for efficient computation

Blood flow dynamics during systolic ejection phase involving fast-moving AV leaflets is known to be highly complex involving strong boundary layer separations and vortical formations. Peak systolic *Re* can range from 4000 to 10 000 based on the degree of AV stenosis [25]. A highly dense mesh (tens of millions) will be required to adequately resolve such complex flow dynamics. Traditionally, this would result in a longer computational time in the order of weeks, even with hundreds of processors [34]. To tackle such challenges efficiently, our Cartesian grid was enhanced with a massively parallel adaptive meshing algorithm with octree smoothing [39]. During the computation process, the mesh will be automatically refined and coarsened locally depending on the velocity and vorticity field gradients (figure 1*g*). A given cell was marked for refinement if $|\omega |h>\varepsilon_{\left|\omega \right|}$ or $|\nabla u|h>\varepsilon_{\left|\nabla u\right|}$ and for coarsening if $|\omega |h<0.25\varepsilon_{\left|\omega \right|}$ or $|\nabla u|h<0.25\varepsilon_{\left|\nabla u\right|}$, where *h* is the characteristic grid sizing and $\varepsilon_{\left|\omega \right|}$ and $\varepsilon_{\left|\nabla u\right|}$ are user-defined tolerances for magnitudes of vorticity (|ω|) and velocity gradients (|∇u|), respectively [39].

The dynamic repartitioning strategy implemented in our solvers has been described in detail elsewhere [39,50]. Briefly, our FSI algorithm has been massively parallelized [34,39], with domain decomposition performed using the open-source partitioning software, ParMETIS [51] with inter-processor communication handled using message passing interface (MPI) libraries [52]. During the solution process, certain parts of the flow domain can undergo significant mesh refinement (e.g. in the vicinity of AV leaflets and Ao during peak systole as shown in figure 1*g*), while others remain unchanged or coarsened (e.g. mid-portions of LV where flow is uniform (figure 1*g*)). The resulting significant load imbalance is tackled by our algorithm using a dynamic repartitioning strategy based upon the current mesh distribution and estimating the amount of computational work. ParMETIS [51] uses this information to create a new partition that results in a new balanced distribution of work (figure 1*h*), thereby increasing the efficiency of computation [39,50]. The combination of these methodologies ensured relatively efficient and accurate FSI simulations that involved highly complex flow dynamics with fast-moving, flexible AV leaflets immersed in a relatively large flow domain with LV and AAo.

## 3. Results

We quantitatively evaluated clinical parameters such as transvalvular pressure gradient, peak systolic velocity and AV area, which are routinely used to assess stenosis severity and post-TAVR performance. We also evaluated leaflet deformation, viscous dissipation [53] and vorticity (identified by Q-criterion) [54] during systolic ejection to provide comprehensive insights into valve performance.

### 3.1. Quantitative comparison with pre- and post-op clinical data

**Figure 2.**
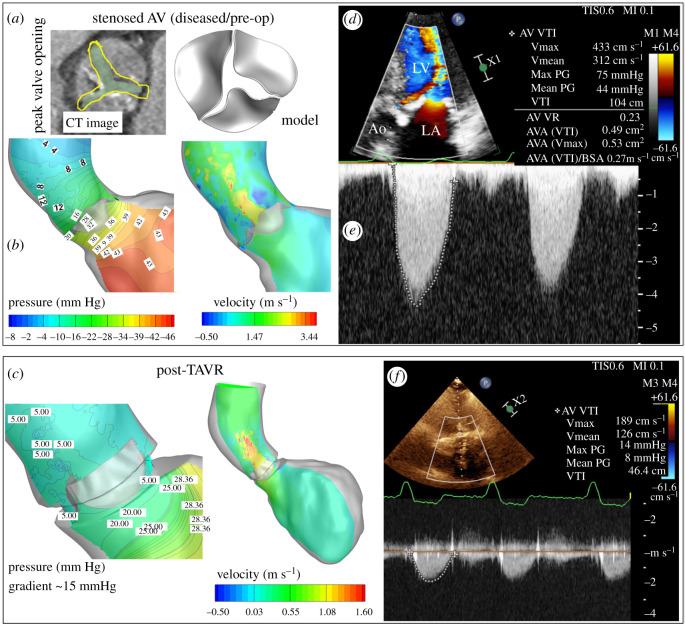
Clinical validation of model predictions with pre- and post-TAVR. (*a*) Comparison of maximum orifice from CT and model prediction for SAV; (*b* and *c*) model predictions of transvalvular pressure gradient and velocity pre- (SAV) and post-TAVR respectively, have a good agreement with (*d*–*f*) corresponding Doppler ultrasound data (three-chamber view) measured pre- and post-TAVR.

Our FSI predictions had good agreement with clinical data (figure 2). The panels of figure 2*a* show the comparison of the model-predicted maximum valve opening of SAV with the corresponding CT image, while the left and right panels of figure 2*b* show transvalvular pressure gradient (approx. 38 mm Hg) and peak systolic velocity magnitude (approx. 3.9 m s^−1^) (electronic supplementary material, figure S1A,B). Model-predicted post-TAVR transvalvular pressure gradients (approx. 15 mm Hg) and peak-systolic velocity magnitude (approx. 1.7 m s^−1^) are shown in figure 2*c* and electronic supplementary material, figure S1A. These pre- and post-TAVR values quantitatively show the degree of improvement achieved post-TAVR and are consistent with clinical image data obtained pre- and post-intervention as a part of the treatment (figure 2*d–f*). Thus, patient-specific FSI models with LV and aorta can potentially predict pre- and post-TAVR dynamics with reasonable accuracy.

### 3.2. Complex LV and Ao flow led to a highly asymmetrical leaflet deformation during systole

**Figure 3.**
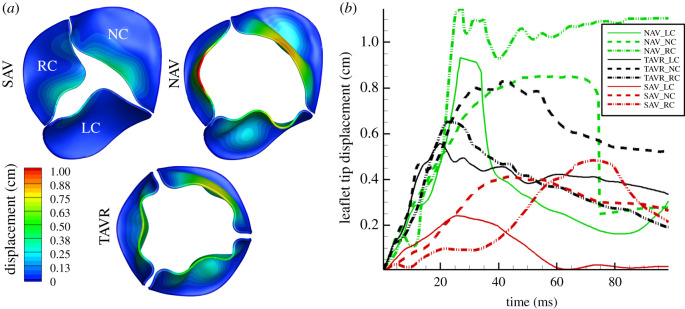
Quantitative evaluation of leaflet displacement; (*a*) maximum AVA predicted in SAV, NAV and TAVR with contour colouring based on displacement in cm. (*b*) maximum leaflet tip displacement (cm) in time (ms) for the first 100 ms when deformation rate is the largest.

To perform a comparative evaluation, all three valve types were subjected to the same LV contraction rate. The increased stiffness in SAV exerted a greater resistance to the oncoming flow from the LV that led to an increased pressure build-up in the left ventricular outflow tract (LVOT) (figure 2*b*). The valve deformation was severely restrained, as shown in electronic supplementary material, movie 1A. The restrained and resistive motion of the leaflets can alter the local flow by changing its angular momentum, as well as the downstream flow (stronger flow separation and roll-up into vortical structures) can ultimately affect individual leaflet dynamics. SAV leaflet deformation was highly asymmetrical during systole, reached a maximum AVA of approximately 1.2 cm^2^ at approximately 36.6 ms (figure 3*a*,*b*) which was consistent with what has been reported in the past for severe aortic stenosis [55]. As LV contraction rate decreased during late systole, the leaflets started moving inwards (electronic supplementary material, movie 1A).

Our model predicted that the TAVR had qualitatively similar but quantitatively different opening dynamics to the NAV. During early systole, NAV and TAVR leaflet deformation led to a triangular-to-circular orifice (electronic supplementary material, movie 1B,C; figure 3*a*). NAV reached its maximum AVA of approximately 3.02 cm^2^ at approximately 52 ms, while TAVR's AVA was predicted to be approximately 2.2 cm^2^ at approximately 29 ms (figure 3*a*) with highly asymmetrical leaflet displacement particularly during early systole (figure 3*b*). During later stages of systole, as flow began to evolve in the sino-tubular junction and LV contraction rate decreased, valve leaflet deformation became asymmetrical and started to move inwards, which is known to aid in efficient closure during diastole (electronic supplementary material, movie 1B,C) [56].

### 3.3. Overall flow dynamics is completely altered post-TAVR

**Figure 4.**
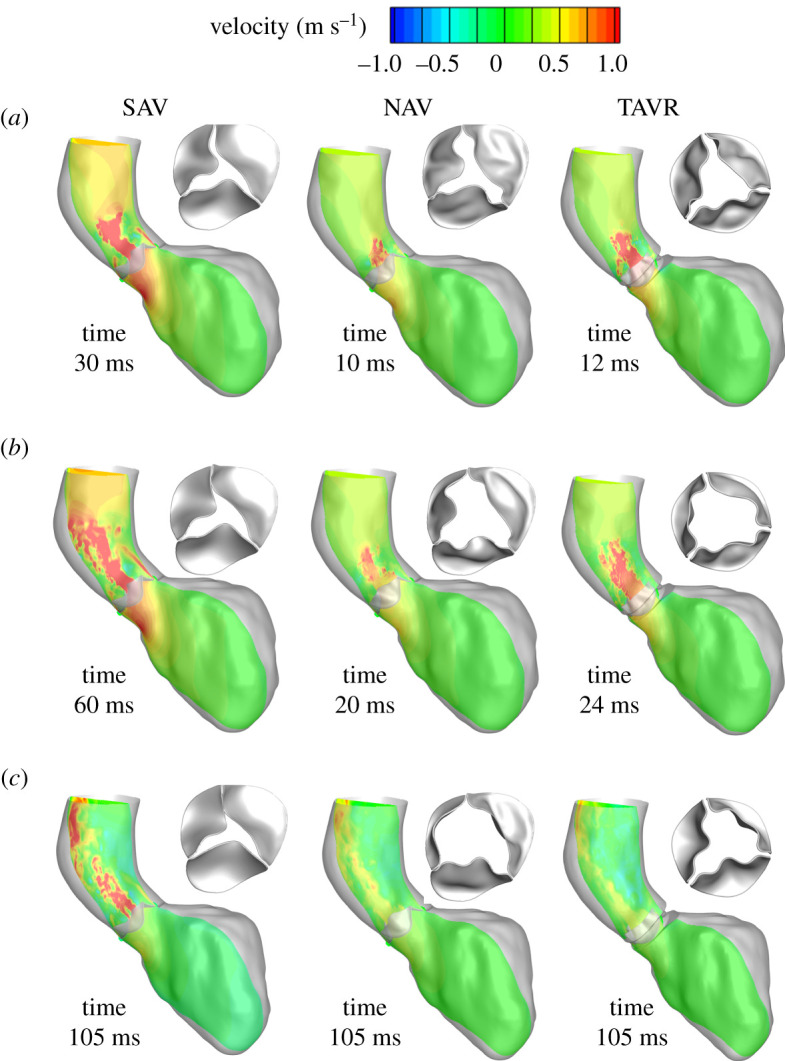
Predictions of overall flow and AV dynamics for SAV (pre-TAVR) (left panel), NAV (middle panel) and post-TAVR (right panel) during (*a*) early, (*b*) peak and (*c*) late systole, show the degree of improvement achieved post-intervention and how it compares with normal AV dynamics.

The increased jet flow for SAV led to a complex flow pattern in the AAo. As the LV continued to contract and due to a narrowed valve opening, the velocity magnitude of the jet emanating from the valve orifice kept increasing and reached a delayed peak systolic velocity of 3.9 m s^−1^ at approximately 60 ms (electronic supplementary material, figure S1A,B). The velocity of the jet seemed well preserved as it impinged on the aortic walls (figure 4*a*–*c* left panel; electronic supplementary material, movie 2A) and subsequently curved upwards towards the aortic arch. On the other hand, due to much wider and faster opening of NAV, the jet was more centralized and reached a peak systolic velocity of approximately 1.14 m s^−1^ at approximately 20 ms (figure 4*b* middle panel and electronic supplementary material, figure S1A,C) which is in the physiological range for normal AVs [57].

It is worthwhile to note that the leaflet deformation of the NAV was highly asymmetrical due to morphology and complex flow dynamics in the LV/LVOT and aorta (figure 3 and electronic supplementary material, movie 1B). The leaflets continued to open, allowing the orifice to further expand (electronic supplementary material, movie 1B), allowing blood flow into the AAo. As the valve reached its maximum orifice area, the velocity magnitude of jet decreased (electronic supplementary material, movie 2B and electronic supplementary material, figure S1A,C). Briefly after staying in a fully open position, the leaflets started to move inwards (figure 4*c* middle panel; electronic supplementary material, movie 2B). Our model predicted a post-TAVR dynamics that looked similar to that of NAV, indicating TAVR's potential in restoring normal aortic flow dynamics (figure 4 right panel; electronic supplementary material, figure S1A,B and electronic supplementary material, movie 2C). However, peak systole was delayed by 4 ms with its velocity at approximately 1.7 m s^−1^ (approx. 1.5-fold greater than NAV) (figure 4*b* right panel; electronic supplementary material, figure S1A).

**Figure 5.**
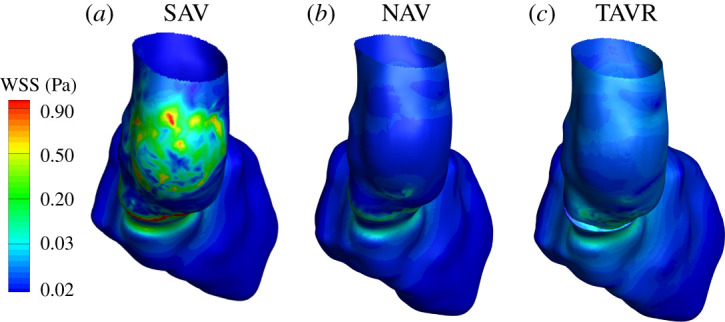
Predictions of wall shear stress (WSS) in Pa for (*a*) SAV (pre-TAVR), (*b*) NAV and (*c*) post-TAVR during peak systole. Posterior view shown with LV apex into the screen. Model predictions show increased WSS in the posterior walls of AAo for SAV, which is decreased post-TAVR.

The shear gradients caused by an increase in velocity magnitude for SAV led to an increase in WSS in posterior AAo (figure 5). The model-predicted maximum WSS of approximately 0.95 Pa near the lateral Ao wall where the jet impinged the AAo was consistent with previous four-dimensional flow magnetic resonance imaging (MRI)-based investigations into local WSS distribution of AAo of patients with aortic stenosis [55,58]. It is also worthwhile to note that WSS is high in the basal LV walls leading up to LVOT. For TAVR and NAV, our model predicted a significantly reduced WSS in AAo (figure 5*b*,*c*). The decrease in local WSS for TAVR (by approx. 3.2-fold) and NAV (by approx. threefold) can be directly attributed to a decreased velocity magnitudes and a relatively efficient opening dynamics that produced a centralized flow for TAVR and NAV (figure 4).

### 3.4. Valve dynamics dictated vortex formation in the ascending aorta

**Figure 6.**
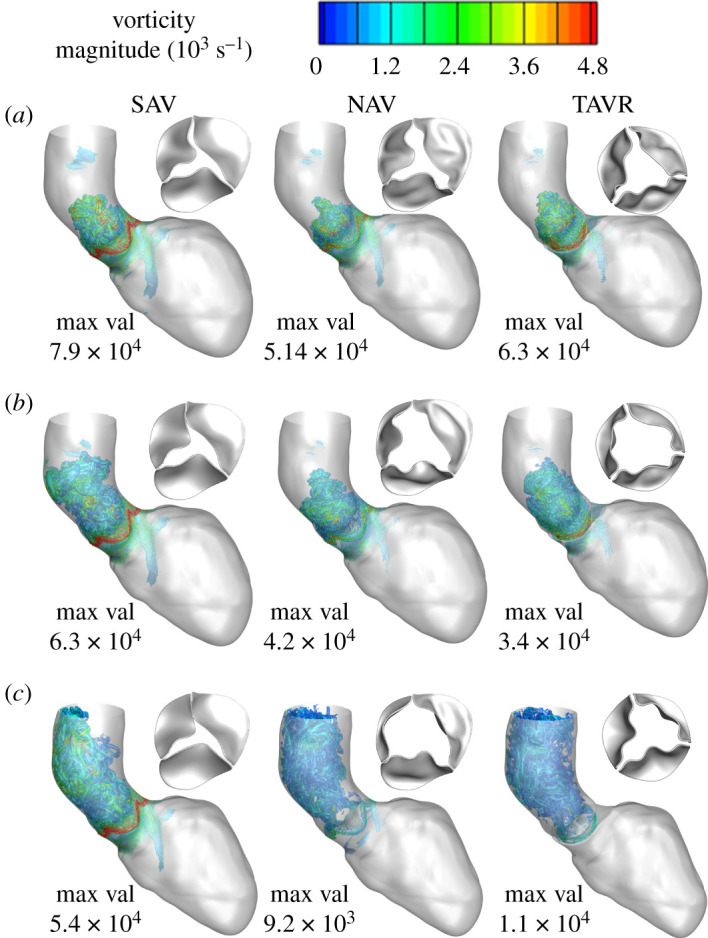
Predictions of vorticity for SAV (pre-TAVR) (left panel), NAV (middle panel) and post-TAVR (right panel) during (*a*) early, (*b*) peak and (*c*) late systole show vorticities peak during early systole and are quantitatively high for SAV while TAVR normalized vorticity in aorta.

The *Re* based on mean flow velocity magnitude (1.04, 0.4 and 0.55 m s^−1^) in the AAo (average diameter 3.3 cm) was calculated to be 10 350, 5600 and 4070 for stenotic SAV, TAVR and NAV, respectively. Based on their respective *Re*s, the flow for both NAV and TAVR was at laminar-to-turbulent transitional regime, while that of SAV was turbulent. As expected for this flow regime, aortic flow patterns were associated with strong boundary layer separations, vortex shedding followed by break-up into smaller incoherent structures that contributed to energy loss. Figure 6 shows comparisons of vortex development for SAV, NAV and TAVR during mid, peak and late systole. Vortex shedding followed by break-up into smaller vortical structures was more intense for SAV in comparison with TAVR and NAV in terms of vortex magnitude (figure 6 and electronic supplementary material, movie 3A–C). Strong vortical structures developed behind the valve in the sino-tubular junction, which moved the valve leaflets toward closure during the early decelerating phase of the systole. For stenotic AV, a vortex ring formed around the strong jet which dissipated into smaller incoherent structures in the AAo.

### 3.5. TAVR reduced viscous dissipation in the aorta during systole

**Figure 7.**
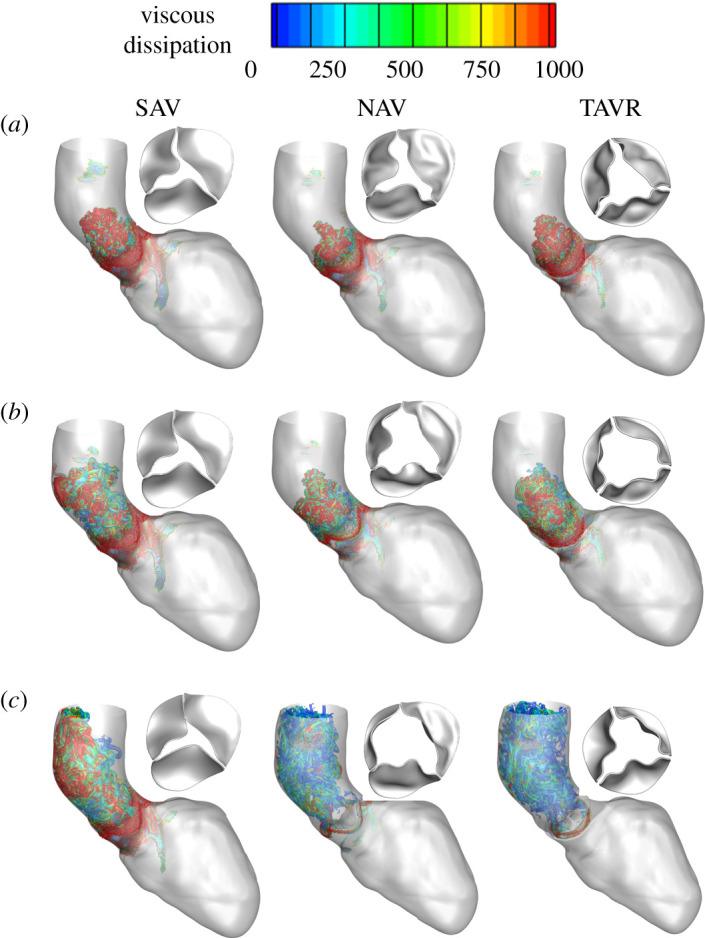
Predictions of viscous dissipation (energy loss) for SAV (pre-TAVR) (left panel), NAV (middle panel) and post-TAVR (right panel) during (*a*) early, (*b*) peak and (*c*) late systole show vorticities peak during early systole and are quantitatively high for SAV. Our model predictions show TAVR can potentially minimize energy loss in aorta.

Vortex shedding and break-ups (figure 6 and electronic supplementary material, movie 3) are directly linked to viscous dissipation and energy loss in the system [53]. Figure 7 and electronic supplementary material, movie 4 show viscous dissipation (energy loss) in the AAo for stenotic AV, TAVR and NAV for mid, peak and late systole, respectively. Due to an increased vortex shedding and subsequent break-up, viscous dissipation was predicted to be the highest for SAV across the systolic phase, particularly during early systole (figure 7 left panel and electronic supplementary material, movie 4A). Our model predicted a significant reduction in viscous dissipation post-TAVR (an average of approximately 2.4-fold during early-to-peak systole and average of approximately 11-fold during late-to-end systole) across the systolic phase that was similar in magnitude to NAV (figure 7 middle and right panel, and electronic supplementary material, movie 4 B and C). These predictions indicate that TAVR can lead to a substantial reduction in energy loss in the aorta. It should also be noted that viscous dissipation was highest during early systole when leaflets undergo a high rate of deformation indicating that valve opening dynamics plays a significant role towards viscous losses in the aorta.

### 3.6. Stenosed AV nearly closed during end systole

**Figure 8.**
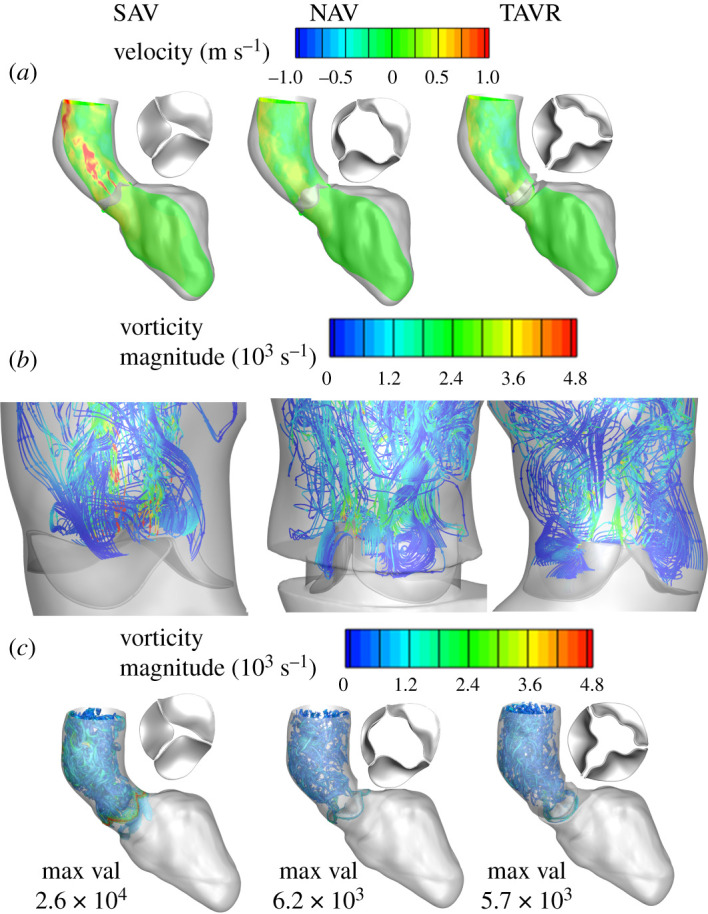
Model predictions of flow dynamics in ascending aorta near end systole (*a*) spatial distribution of velocity and valve configuration; (*b*) re-circulation behind AV leaflets with stream traces coloured based on vortex magnitudes; and (*c*) vorticity coloured based on its magnitude, of SAV, NAV and TAVR, respectively.

The AV leaflets kept moving towards closure until end systole (approx. 210 ms) (figure 8*a*,*b* and electronic supplementary material, movie 1). This was due to the combination of (i) decrease in the LV contraction rate and (ii) flow reversal behind the leaflets pushing the leaflets toward closure (figure 8*b*). Model predictions showed that the effect was greatest for SAV which closed nearly completely during end-systole (figure 8*a* left panel) followed by TAVR (figure 8*a* right panel) and NAV (figure 8*a* middle panel) in that order. As SAV moved towards complete closure, a jet, albeit of weaker magnitude, was seen emanating from the constricted orifice (figure 8*a* left panel; electronic supplementary material, figure S1A,B) that contributed to the complex vortex evolution with higher vortex magnitude in the AAo when compared with NAV and TAVR (figure 8*c*).

## 4. Discussion

In this study, we implemented a new modelling framework for evaluating patient-specific AV dynamics, left ventricular and aortic flow as a high-fidelity FSI problem using previously developed and validated algorithms with parallel computing capability (figure 1 bottom panels). A level set-based sharp interface Cartesian grid with flow-based local mesh refinement capability was used to accurately capture the highly complex aortic blood flow dynamics during the systolic ejection phase [34,39,50] (figure 2). For the solid solver, the AV leaflets were discretized as eight-node EAS elements which are known to have superior bending accuracy with minimal locking effects [28,29], and its capability to model thin and pliant heart valve structure was demonstrated in our previous mitral valve study [34]. The fluid and solid solvers were tightly coupled by means of sub-iterative loops with dynamic Aitken under-relaxation for numerical stability and accelerated convergence [34,44,45] (figure 1 bottom panel). The computational domain was dynamically repartitioned for efficiency during simulation based on the computational load determined by the local mesh distribution (figure 1*g*,*h*).

We used this modelling framework to develop a novel workflow for evaluating post-op TAVR performance against patient's virtually decalcified native AV (patient-specific benchmark) and calcified (diseased) valve to quantify the degree of improvement achieved post-TAVR under physiologically realistic flow conditions (figure 1 top panel). A retrospective study was done using clinical data from a patient who underwent a 23 mm TAVR for a severely SAV. Our framework allowed us to achieve highly resolved laminar-to-turbulent systolic flow dynamics in the LV and ascending aorta (AAo) with approximately 15 million cells at the start of the simulation, with our LMR algorithm refining the computational domain locally up to approximately 19 million during the simulation (electronic supplementary material, figure S2). One-hundred ninety-two central processing units (CPUs) were employed for each of the three cases with a wall clock time of approximately 48 h.

Our model-predicted peak systolic dynamics had a good correlation with pre- and post-TAVR clinical data (figure 2). The valve deformation predicted for the SAV also had a good qualitative agreement with CT data (figure 2*a*). Our model thus was able to accurately predict the physiologically realistic fluid dynamics in the AAo using clinical data. Model predictions of valve deformation, AV area, velocity, transvalvular pressure, wall shear stress, vorticity and viscous dissipation function allowed comprehensive evaluation of valvular function and its associated flow dynamics. These evaluation parameters were used to quantitate diseased AV and post-TAVR dynamics and to predict how post-TAVR restored normal flow by comparing it against the hypothetical benchmark of virtually de-calcified patient's native AV. Such FSI-based modelling frameworks incorporating patient-specific data acquired during clinical diagnosis can offer predictive insights on post-TAVR function and haemodynamics with reasonable accuracy towards choosing the right TAVR type and size and leaflet orientation to improve long-term success.

AV stenosis is not just limited to AV leaflets becoming stiffer affecting the blood flow around it but affects both the LV and the downstream systemic vasculature [27]. Immediately downstream, aortic dilatation is common (note that the patient in the present study had a mildly dilated AAo with a diameter of approx. 3.6 cm) that may also need to be addressed at the time of intervention [27,59]. Our model predicted distinctly different ejection dynamics in the SAV compared with the other two valves (figures 3–8, and electronic supplementary material, movies 1–4) with a *Re* of 10 000 in the aorta consistent with previously reported values [25]. Peak systolic *Re* in SAV was approximately twice that of TAVR and NAV. The LMR algorithm was able to resolve the complex flow pattern which involved high-speed jet flows, incoherent vortical structures, which are typical for such high *Re* flows (figures 3–8, and electronic supplementary material, movies 1–4).

The narrowed valve opening of SAV produced a strong jet that impinged on aortic walls (figure 4*a*–*c* left panel and electronic supplementary material, movie 2A). High velocity flows (electronic supplementary material, figure S1A,B) characterized by high shear gradients (effectively captured by the highly dense local mesh) in SAV led to distinctively high magnitudes of WSS in the AAo (figure 4*a*). WSS is a known mechanotransduction stimulus that can impact cell function and influence aortic wall remodelling [60,61] that has been confirmed by both four-dimensional MRI and histopathology-based studies [58,62]. The elevated WSS in the AAo for SAV (figure 5*a*) may have contributed to the aortic wall dilation observed in our patient. Similarly, on the upstream side, AV stenosis is associated with LV remodelling and hypertrophy in the initial stages followed by LV decompensation in the later stages driven by myocardial fibrosis [27,63,64]. Inclusion of patient-specific LV and aortic geometry in the modelling framework can potentially contribute to predictive accuracy and provide additional insights into the effect of SAV-induced abnormal flow on adjacent structures.

Well-formed vortex is thought to preserve momentum and kinetic energy, while disturbed vortex formation is associated with significant energy loss. MRI-based studies have shown that viscous energy losses were elevated in patients with disturbed vortex ring formation during LV filling [53] and in the aorta of patients with SAVs [65]. Our model predictions showed a highly disturbed vortex in the aorta of SAV with magnitudes approximately fourfold greater than NAV and TAVR (figure 6*c*) and had direct correlation with viscous dissipation (figure 7). It should be noted that viscous dissipation substantially reduced post-TAVR and was comparable to NAV suggesting that aortic blood flow efficiency is potentially restored post-TAVR. Our results suggest that analysis of vortex and viscous dissipation provides additional quantitative insights into aortic blood flow post-AV intervention.

Even though our models captured essential valvular function and blood flow dynamics that had a good agreement with the clinical data, our study had the following limitations. LV contraction was modelled using a spatially and temporally varying Gaussian function. Although it captured the end-diastolic and end-systolic LV volumes in time, it may not have fully captured the exact patient-specific LV contraction. To tackle this limitation, our model is being refined to map model-predicted LV contraction spatially and temporally with that of clinical data. The effect of valve stenosis was represented by increasing leaflet material stiffness, thus ignoring local geometric changes predominantly by calcium deposits. Efforts are underway to include realistic calcium deposits on valve surfaces during computations. In the present study, the interaction between native calcified AV and TAVR was ignored. Hence, the biomechanical interplay between TAVR device and the aortic landing zone could not be captured. Efforts are underway to include native AV-TAVR interaction to quantify paravalvular leakage and predict device migration. Next, we used leaflet material properties for native as well as TAVR leaflets from the literature [30–32]. Material properties for native valves vary with patients, and obtaining patient-specific material properties is not feasible using current diagnostic techniques. Aortic deformation during systolic opening of AVs was not included but will be accounted for in future models. Our three-dimensional model reconstruction involved semi-automated and manual editing methods for LV, AV and Ao. Careful identification of calcium deposits followed by virtual decalcification and valve reconstruction required time (approx. 50 h) that could impact clinical timeframe for TAVR planning as well as the geometric accuracy. Machine learning-based valve segmentation and reconstruction methods [66] that are being developed offer a viable solution towards reducing the total time for three-dimensional reconstruction.

## 5. Conclusion

We have demonstrated a novel workflow that offers comprehensive predictive insights for evaluating AV performance and benchmarking an implanted valve against a virtually de-calcified normal valve. We used our workflow to evaluate the performance of a 23 mm TAVR to predict the degree of improvement achieved post-TAVR using patient-specific modelling. Our model predictions included valvular function and flow dynamics in the LV and in the aorta, parameters such as transvalvular pressure gradients and peak systolic velocity that are routinely used to access valve performance clinically. We added parameters such as aortic WSS, vorticity and viscous dissipation to determine valve functional characteristics more comprehensively. Our results provided quantitative insights into the implications of AV stenosis in the Ao and LV pre- and post-TAVR and how they compare with patients' hypothetical normal AV. This workflow can help determine optimal valve choice and position, and predict potential complication prior to TAVR or surgical AV replacement.

## Data accessibility

Our study used patient-specific three-dimensional geometry of left ventricle, ascending aorta and native aortic valve leaflets reconstructed from patient's CT image data. The three-dimensional reconstructions and their mesh files used in this study are available in the GitHub repository: https://github.com/gvjgvj/TAVRFSIAnalysis and have been archived within the Zenodo repository: doi:10.5281/zenodo.5802030. Readme file provided along with the data provides brief instructions on how to read/access/use the data. The provided data does not contain any protected health information abiding by HIPAA rules for human subject's protection.
